# A prospective cohort study providing insights for markers of adverse pregnancy outcome in older mothers

**DOI:** 10.1186/s12884-021-04178-6

**Published:** 2021-10-20

**Authors:** Samantha C. Lean, Rebecca L. Jones, Stephen A. Roberts, Alexander E. P. Heazell

**Affiliations:** 1 Maternal and Fetal Health Research Centre, Division of Developmental Biology and Medicine, Faculty of Biology, Medicine and Health, University of Manchester, St. Mary’s Hospital, 5th Floor (Research), Oxford Road, Manchester, M13 9WL UK; 2 Centre for Biostatistics, Faculty of Biology, Medicine and Health, University of Manchester, Manchester, UK

**Keywords:** Aging, Biomarkers, Hormones, Inflammation, Oxidative stress, Stillbirth, Placental dysfunction

## Abstract

**Background:**

Advanced maternal age (≥35 years) is associated with increased rates of adverse pregnancy outcome. Better understanding of underlying pathophysiological processes may improve identification of older mothers who are at greatest risk. This study aimed to investigate changes in oxidative stress and inflammation in older women and identify clinical and biochemical predictors of adverse pregnancy outcome in older women.

**Methods:**

The Manchester Advanced Maternal Age Study (MAMAS) was a multicentre, observational, prospective cohort study of 528 mothers. Participants were divided into three age groups for comparison 20–30 years (*n* = 154), 35–39 years (*n* = 222) and ≥ 40 years (*n* = 152). Demographic and medical data were collected along with maternal blood samples at 28 and 36 weeks’ gestation. Multivariable analysis was conducted to identify variables associated with adverse outcome, defined as one or more of: small for gestational age (< 10th centile), FGR (<5th centile), stillbirth, NICU admission, preterm birth < 37 weeks’ gestation or Apgar score < 7 at 5 min. Biomarkers of inflammation, oxidative stress and placental dysfunction were quantified in maternal serum. Univariate and multivariable logistic regression was used to identify associations with adverse fetal outcome.

**Results:**

Maternal smoking was associated with adverse outcome irrespective of maternal age (Adjusted Odds Ratio (AOR) 4.22, 95% Confidence Interval (95%CI) 1.83, 9.75), whereas multiparity reduced the odds (AOR 0.54, 95% CI 0.33, 0.89). In uncomplicated pregnancies in older women, lower circulating anti-inflammatory IL-10, IL-RA and increased antioxidant capacity (TAC) were seen. In older mothers with adverse outcome, TAC and oxidative stress markers were increased and levels of maternal circulating placental hormones (hPL, PlGF and sFlt-1) were reduced (*p* < 0.05). However, these biomarkers only had modest predictive accuracy, with the largest area under the receiver operator characteristic (AUROC) of 0.74 for placental growth factor followed by TAC (AUROC = 0.69).

**Conclusions:**

This study identified alterations in circulating inflammatory and oxidative stress markers in older women with adverse outcome providing preliminary evidence of mechanistic links. Further, larger studies are required to determine if these markers can be developed into a predictive model of an individual older woman’s risk of adverse pregnancy outcome, enabling a reduction in stillbirth rates whilst minimising unnecessary intervention.

**Supplementary Information:**

The online version contains supplementary material available at 10.1186/s12884-021-04178-6.

## Introduction

Advanced maternal age (≥35 years) is a growing trend in high income countries [1, 2]. Large epidemiological studies and subsequent meta-analysis have identified maternal age ≥ 35 years as an independent risk factor for adverse fetal outcomes including: fetal growth restriction (FGR), pre-term birth (PTB), pre-eclampsia (PE), neonatal intensive care unit (NICU) admission and stillbirth [3–8]. Stillbirths in older women are likely to occur near term with risks comparable to those in obesity, smoking, diabetes or history of stillbirth [9–11]. However, unlike these conditions there are few guidelines to reduce adverse outcomes in older women [12, 13].

There is international recognition that older women should undergo additional antepartum screening or intervention to address the increased risk of stillbirth [14–17]. The RCOG and SOGC recommend offering induction of labour (IOL) at 39 weeks and/or additional monitoring from 38 weeks’ gestation [2, 15, 18]. Although not associated with an increase in the rate of Caesarean section [19], IOL may be viewed as an unnecessary intervention as the majority of mothers will have uncomplicated pregnancies. Furthermore, induction may not be an acceptable intervention for older women without further indication, with poor recruitment (only 13.6%) of eligible women to the 35–39 trial consenting to be randomised [19]. Identification of mothers with highest risk would result in fewer interventions to prevent stillbirths.

Many pregnancy pathologies are associated with changes in oxidative stress and inflammatory status [20–22]. Similar changes are reported in aging processes although these are usually researched in older populations [23, 24]. If these alterations were present in older women they could adversely affect placental function [25–27]. Previous work found evidence of placental dysfunction in pregnancies in older women including (but not limited to) reduced amino acid transport, aberrant cell turnover and reduced placental efficiency [28]. Therefore, it was hypothesised that the increased risk of adverse pregnancy outcome results from an aging maternal environment and that a combination of biomarkers of aging, placental dysfunction, and clinical risk factors might identify women at greatest risk. This study aimed to determine whether there were changes in oxidative stress and inflammation in pregnancies in older mothers and whether changes in these biomarkers were evident in adverse pregnancy outcomes in this population.

## Methods

Women were recruited to the Manchester Advanced Maternal Age Study (MAMAS) from March 2012–October 2014 from six UK maternity units after providing written informed consent. Ethical approval was obtained from the NRES Committee North West, (12/NW/0015). Pregnant women aged between 20 and 30 years (controls – optimal reproductive age), 35–39 years and ≥ 40 years were approached at 28 weeks’ gestation between April 2012–June 2014. Women with multiple pregnancy, body mass index (BMI) < 18.5 or > 30 kg/m^2^, fetal abnormalities, and pre-existing maternal medical conditions were excluded.

In addition to usual antenatal care, participants attended prenatal research appointments at 28 and 36 weeks’ gestation (±1 week), at which detailed demographic, medical data and maternal blood samples for plasma and serum fractionation were collected. After delivery, outcome data were collected from medical records. Biochemical analyses were conducted after delivery, therefore not altering participants’ prenatal care.

The Index of Multiple Deprivation (IMD) – a measure of relative social deprivation [29] - was calculated from the mother’s address using NPEU-IMD tool (University of Oxford, UK). A composite adverse pregnancy outcome was defined as one or more: small for gestational age (SGA) or FGR (< 10th/<5th centile respectively using individualised birthweight centiles (IBC) [30], stillbirth, admission to the NICU, PTB without infection (< 37 weeks gestation), and 5 min Apgar score < 7 in the absence of maternal diseases (diabetes/hypertension). Normal pregnancy outcome was defined as a term live birth (38–42 weeks), appropriately grown (IBC between 10-95th centile) and absence of maternal or fetal complication (not limited just to those included in our definition of adverse pregnancy outcome). We conservatively estimated a 20% incidence of adverse pregnancy outcome (using data from Reference [8] stillbirth rate 0.4%, incidence of FGR 6%, neonatal unit admission 8%, preterm birth 10%), therefore approximately 600 participants were required to obtain 120 women with composite adverse outcome; 120 women with adverse outcome would allow multivariable analysis of six covariates.

### Nested case control studies

Two nested case-control studies were conducted i) to determine whether pregnancy in older mothers associated with elevated circulating biomarkers of inflammation and oxidative stress and ii) to determine whether adverse pregnancy outcome was associated with markers of aging and placental dysfunction in women ≥35 years of age. In the first nested case-control study samples from participants ≥40 years were matched (1:1) to mothers aged 20–30 and 35–39 years (*n* = 40/group) for demographic (BMI, IMD, ethnicity (groups), marital status (married, single, partnership), smoking status (current/ex/non-smoker) and obstetric characteristics (normal vaginal delivery (NVD)). Women with adverse pregnancy outcome, who conceived via assisted reproductive technology (ART) or in whom maternal disease that developed post 28 weeks’ gestation were excluded. Sample sizes were determined following power calculations based on previous studies for detecting a difference in circulating oxidative stress [31–34] and inflammatory mediators [35–37]. For example, to detect a difference in cytokines TNF-α and IL-6 between normal and adverse outcomes in older women with 80% power with a 5% significance level required between 28 and 43 participants in each group.

For the second nested case control study, cases were 43 women ≥35 years of age who had an adverse outcome as defined above. Controls were participants with maternal age ≥ 35 years with a normal pregnancy outcome. Participants that had maternal disease that developed post 28 weeks, PTB associated with infection, or incomplete outcome data were excluded. Groups were matched for demographic characteristics (maternal/paternal age, ethnicity, BMI, smoking status, marital and housing (ownership/rental) status and parity (primi/multiparous.

Biomarkers of aging were measured in maternal serum or plasma samples for participants included in the nested case control studies (Supplementary Table 1). Absorbances were measured using a microplate reader (FLUOStarOmega, BMG Labtech) for all Enzyme linked immunosorbent assays (ELISAs). Pro/anti-inflammatory cytokines (interleukin (IL)-1α, IL-1β, IL-1Ra, IL-6, IL-10 and tumour necrosis factor (TNF)-α) were quantified in maternal serum using DuoSet®ELISAs (R&D Systems, Abingdon, UK) following an optimised protocol [27].

Maternal plasma antioxidant concentration was quantified using OxiSelect™ Total Antioxidant Capacity Assay Kit (Cell Biolabs, Inc., San Diego, USA). Oxidative damage markers 8-Isoprostane and DNA/RNA oxidative damage were measured by EIA Kits and Protein Carbonyl Calorimetric Assay Kit (Cayman Chemical Company, Michigan, USA). Maternal serum placental hormones (hCG, PAPP-A, Progesterone and hPL were quantified using DRG ELISA kits (DRG Instruments, Marburg, Germany). Placental growth factor (PlGF) and soluble fms-like tyrosine kinase-1 (sFlt) were quantified using DuoSet® ELISAs (R&D systems). All assays were conducted according to the manufacturer’s standard protocols.

### Statistical analysis

Demographic data were compared using Fisher’s Exact test for categorical data and Kruskal-Wallis tests with Dunn’s multiple comparisons or Mann Whitney U tests for continuous data. Univariate logistic regression was conducted on the whole dataset to identify demographic or clinical variables associated with composite adverse pregnancy outcome. Multivariable logistic regression was used to quantify the effect of maternal age on adverse outcome; in this analysis maternal ethnicity, use of ART, smoking status, parity, IMD and home ownership were included as categorical variables and in an second model, paternal age was included as an additional categorical variable (< 30, 30–34, 35–39 and 40+ years). The logistic regression analyses were conducted using STATA (Version 14, StataCorp, Texas, USA). Biomarker analysis was performed on GraphPad Prism (Version 6.04, GraphPad Software Inc., La Jolla, USA) using Kruskal-Wallis with Dunn’s multiple comparisons tests on untransformed data or Mann-Whitney U where appropriate. Due to wide dispersion in the cytokine data, including values at the lower limit of detection of the assay, these data were transformed as log(y + smallest detectable value) for analysis. Gestational age effects were assessed using Spearman’s correlation. Markers that showed statistical significance at the *p* < 0.01 level were analysed to test their predictive potential as markers of adverse pregnancy outcome in women aged ≥35 years by calculation of the area under the receiver operator characteristic curve (AUROC).

## Results

**Fig. 1:**
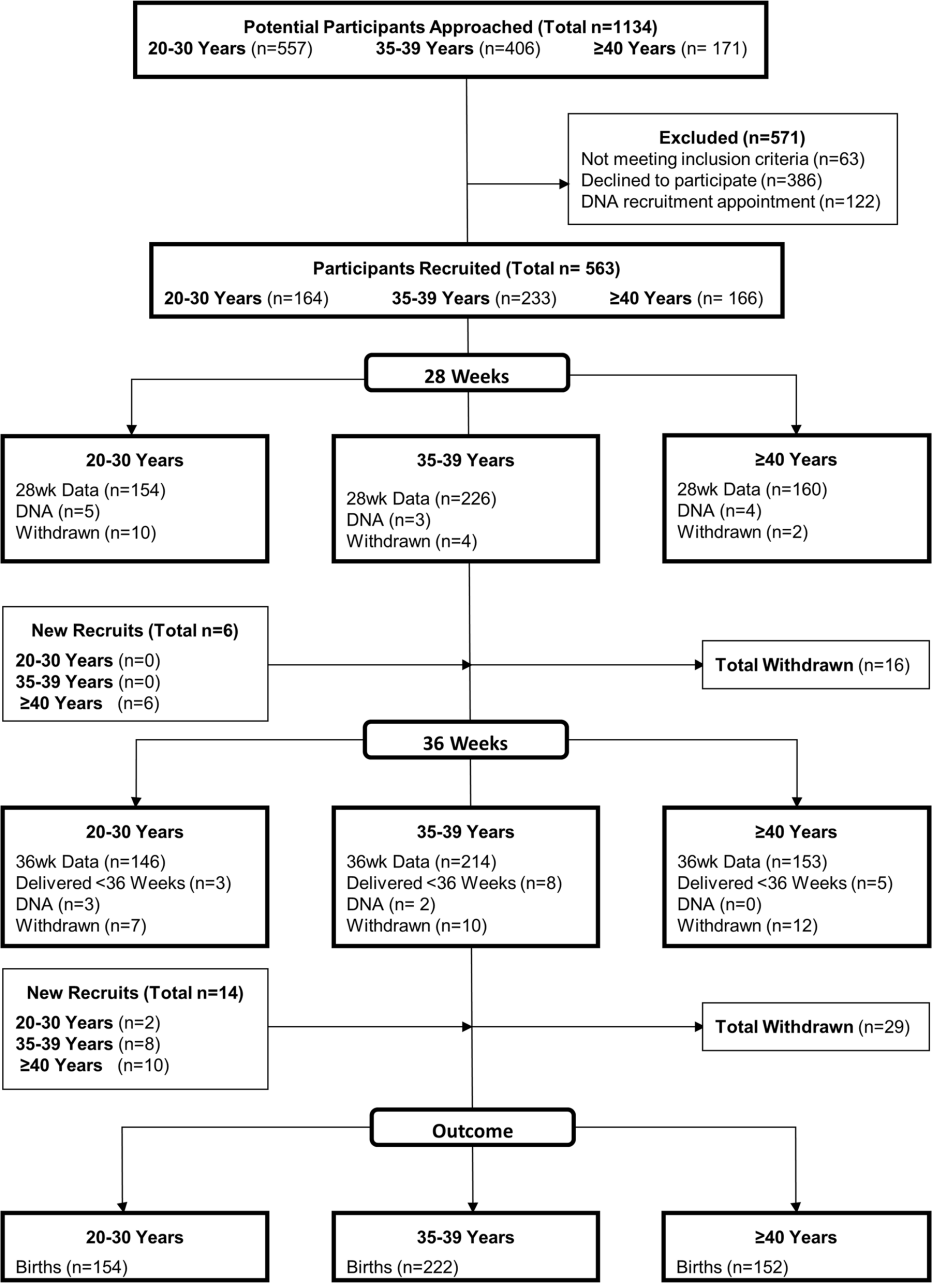
Flow diagram of participant recruitment and retention in the MAMAS cohort study

Overall, 1134 women were approached to participate in MAMAS. 571 of these mothers (51%) either did not meet the inclusion criteria or declined to participate (Fig. 1). 563 mothers consented before 28 weeks’ gestation. A further 45 participants were recruited between 28 weeks’ gestation and birth. 80 mothers (14%) were either lost to follow up or withdrew from the study between 28 weeks’ gestation and birth. Therefore, demographic, medical and pregnancy data was collected on a final cohort of 528 participants (*n* = 154 20–30, *n* = 222 35–39 and *n* = 152 ≥ 40 year olds).

There were no differences between ethnicity, employment and deprivation between participants in different age groups (Table 1). Paternal age was higher in pregnancies to women aged 35–39 and ≥ 40 years, and women in both these groups had higher BMI compared to 20–30 year olds. More mothers aged 35–39 years were married, and more mothers aged 20–30 years were in partnerships than the other maternal age groups. More older mothers were non-smokers, homeowners, multiparous and had previous or current fertility treatment (predominantly in vitro fertilisation) compared to women aged 20–30. Of those who conceived using assisted reproductive technologies, 45% of those ≥40 years used egg and/or sperm donors, compared to 5% of 35–39 year olds.

**Table 1:** Demographic data for total MAMAS participants

Demographics	20–30 Years (***n*** = 154)	35–39 Years (***n*** = 222)	≥40 Years (***n*** = 152)	***P*** value Overall	***P*** value Multiple Comparisons
Maternal age	**26** (20–30)	**37** (35–39)	**42** (40–49)		
Paternal Age ^a^	**29** (18–48)	**38** (21–50)	**43** (24–60)	**< 0.001**	^†¶Δ^ **< 0.0001**
BMI (kg/m^2^) ^a^	**23.8** (18.7–29.9)	**24.1** (18.5–29.5)	**24.9** (19.0–29.9) 1 missing value	**0.0026**	^†^0.10, ^¶^**0.0004**, ^Δ^**0.008**
European Ethnicity^b^	**90%** (138)	**87%** (191)	**95%** (144)	0.08	
Marital Status ^b^					
*Single*	**5%** (7)	**2%** (5)	**5%** (8)	**0.001**	^†<^ **0.001**, ^¶^0.15, ^Δ^0.08
*Married/CP*	**45%** (69)	**66%** (146)	**55%** (83)		
*Partner*	**51%** (78)	**31%** (69)	**39%** (60)		
*Other*	**0%** (0)	**1%** (1)	**1%** (1)		
Occupation ^b^					
*Employed – Full Time*	**55%** (84)	**49%** (109)	**59%** (89)	0.51	
*Employed – Part Time*	**26%** (40)	**32%** (72)	**26%** (40)		
*Unemployed*	**18%** (28)	**16%** (35)	**14%** (21)		
*Unknown*	**1%** (2)	**3%** (6)	**1%** (2)		
Smoking Status ^b^					
*Non-smoker*	**71%** (110)	**74%** (165)	**81%** (123)	0.056	
*Current*	**11%** (17)	**5%** (12)	**3%** (5)		
*Ex-smoker*	**18% (**27)	**20%** (45)	**16%** (24)		
Housing ^b^					
*Owns*	**45%** (70)	**73%** (163)	**84%** (127)	**< 0.001**	^†¶^ ***p*** **< 0.001,**^Δ^**0.023**
IMD ^a^	**19.09**	**16.11**	**15.54**	0.25	
*Score*	(2.28–71.95) 4 missing values	(1.94–76.09) 7 missing values	(1.94–69.63) 13 missing values		
Parity ^b^					
*Primiparous*	**49%** (76)	**28%** (62)	**34%** (52)	**< 0.001**	^†^ < **0.001**, **0.001**, ^Δ^0.092
*Parous*	**51%** (86)	**72%** (160)	**66%** (100)		
Previous APO ^b,c^	**22%** (17)	**32%** (51)	**22%** (22)	0.12	
*FGR* ^c^	**13%** (10)	**21%** (33)	**11%** (11)	0.09	
*Stillbirth* ^c^	**0%** (0)	**3%** (4)	**4%** (1)	0.52	
Previous ART ^b^	**< 1%** (1)	**8%** (18)	**18%** (28)	**< 0.001**	^†^**0.001**, ^¶^ < **0.001**, ^Δ^**0.004**
ART ^b^	**< 1%** (1)	**9%** (21)	**14%** (22)	**0.004**	^†^**0.001**, ^¶^ < **0.001**, ^**Δ**^**0.005**
*Hormonal*	**< 1%** (1)	**2%** (5)	**1%** (2)		
*IVF*	**0%** (0)	**6%** (13)	**7%** (10)		
*IVF - Donor*	**0%** (0)	**< 1%**(1)	**7%** (10)		
*IUI*	**0%** (0)	**1%** (2)	**0%** (0)		

Data are mean (range) or percentage (number). *BMI* Body mass index, *CP* Civil partnership, *IMD* Index of multiple deprivation, *APO* Adverse pregnancy outcome, *ART* Assisted reproductive therapies, *IVF* In vitro fertilisation, Donor = egg and/or sperm, *IUI* Intrauterine insemination. Statistical differences are ^a^Kruskal-Wallis with Dunn’s multiple comparisons or ^b^ Fishers exact test. ^c^ expressed as percentage of parous women. When overall *p* > 0.05, multiple comparisons *p* values are reported (^†^ 20–30 vs. 35–39 years, ^¶^ 20–30 vs ≥40 years, ^Δ^ 35–39 vs. ≥40 years). Significant differences are highlighted with bold *p* values

Older women delivered moderately earlier than women aged 20–30 years (39 weeks + 3 days vs. 40 weeks + 2 days; Table 2). Women ≥40 years had a 43% rate of IOL compared to 26–32% in the younger groups (*p* = 0.002). Fewer older mothers had NVDs (39% vs 50 and 60%) *p* = 0.001) and more had elective or emergency caesarean sections. Birthweight, IBC and the incidence of pregnancy-related maternal disease did not differ between the maternal age groups. Four women in MAMAS had a stillbirths (between 38^+ 1^ and 40^+ 3^ weeks gestation), all were ≥ 40 years old (*p* = 0.007).

**Table 2:** Pregnancy outcome of MAMAS participants

Pregnancy Outcome	20–30 Years (***n*** = 154)	35–39 Years (***n*** = 222)	≥40 Years (***n*** = 152)	***P*** value Overall	***P*** Value Multiple Comparisons
Gestation at Delivery ^a^	**40 + 2**	**39 + 4**	**39 + 3**	**< 0.001**	^†^**0.003**, ^¶^ **< 0.0001**, ^**Δ**^**0.04**
Weeks + days	(34 + 1–42 + 5)	(39 + 6–43 + 1)	(30 + 5–42 + 4)		
Birthweight (g) ^a^	**3416** (1880–4680)	**3438** (1300–4900)	**3375** (1480–4420)	0.94	–
Individualised Birthweight Centile ^a^	**38.4** (0.1–98.7)	**45.3** (0.0–99.6)	**47.7** (0.0–99.4)	0.35	–
Induction of Labour ^b^	**32%** (50)	**26%** (58)	**43%** (66)	**0.002**	^†^0.20, ^¶^0.06, ^Δ^ **0.001**
Mode of Birth ^b^					
*Normal Vaginal Delivery*	**60%** (102)	**50%** (117)	**39%** (64)	**< 0.001**	^**†**^ **0.001,**^**¶**^ **< 0.001,** ^Δ^ 0.17
*Elective Caesarean Section*	**13%** (12)	**25%** (47)	**25%** (35)		
*Emergency Caesarean Section*	**8%** (12)	**10%** (25)	**17%** (26)		
*Instrumental Vaginal Delivery*	**19%** (28)	**15%** (33)	**19%** (27)		
Maternal Disease ^b^					
*Preeclampsia*	**5%** (8)	**1%** (2)	**3%** (4)	**0.04**	^**†**^ **0.01,**^**¶**^ 0.38**,** ^Δ^ 0.23
*Gestational Diabetes Mellitus*	**1%** (2)	**3%** (7)	**3%** (4)	0.56	
NPO ^b^	**77%** (119)	**81%** (180)	**80%** (121)	0.67	
APO ^b^	**23%** (35)	**19%** (42)	**20%** (31)	0.69	
Components of APO					
*Pre-Term Birth*	**6%** (9)	**6%** (13)	**6%** (9)	1.00	
*Small for gestational Age*	**10%** (15)	**11%** (25)	**9%** (14)	0.73	
*Fetal Growth Restriction*	**3%** (5)	**7%** (16)	**4%** (6)	0.21	
*Large for Gestational Age*	**3%** (4)	**5%** (12)	**4%** (6)	0.40	
*Neonatal Unit Admission* ^*c*^	**4%** (6)	**7%** (15)	**7%** (11)	0.36	
*Stillbirth*	**0%** (0)	**0%** (0)	**3%** (4)	**0.007**	^†^1.00, ^¶^0.06, ^Δ^ **0.03**

Data are mean (range) or percentage (number). Statistical differences are ^a^Kruskal-Wallis with Dunn’s multiple comparisons or ^b^ Fishers exact test. ^c^ Expressed as proportion of babies born alive. When overall *p* > 0.05, multiple comparisons *p* values are reported (^†^ 20–30 vs. 35–39 years, ^¶^ 20–30 vs ≥40 years, ^Δ^ 35–39 vs. ≥40 years)

Demographic predictors of adverse pregnancy outcome: Univariate logistic regression demonstrated that ex- and current smokers had higher odds of adverse pregnancy outcome than non-smokers (OR 1.96 (95%CI 1.17–3.30) and 3.97 (95%CI 1.92–8.21), *p* = 0.01 and < 0.001 respectively; Table 3). Multiparity was protective against adverse pregnancy outcome (OR 0.67 (95%CI 0.44–1.03), *p* = 0.05) compared to primiparous women. In this population, maternal ethnicity did not independently affect the risk of adverse pregnancy outcome. ART had no detectable effect on outcome (OR 0.94 (95%CI 0.44–2.01) *p* = 0.88). Paternal age had a protective effect against adverse outcome (OR 0.54 (95%CI 0.31–0.94) *p* = − 0.03). Only the associations between adverse pregnancy outcome and maternal parity or cigarette smoking remained statistically significant after adjusting for maternal ethnicity, parity, smoking status, housing, and paternal age (Table 3). Following adjustment for covariates maternal age ≥ 40 was associated with increased risk of adverse perinatal outcome (AOR 2.67, 95% CI 1.09–6.52, *p* = 0.03).

**Table 3:** Unadjusted and adjusted odds ratios for prediction of adverse pregnancy outcome of MAMAS participants

	Normal Outcome (***n*** = 420) (N(%))	Adverse Outcome (***n*** = 108) (N(%))	Unadjusted		Model 1		Model 2	
			OR (95% CI)	***P value***	AOR (95% CI)	***P value***	AOR	***P value***
Ethnicity								
*European*	373 (89)	101 (94)	Reference		Reference		Reference	
*Asian*	24 (6)	5 (5)	0.77 (0.29–2.07)	*0.49*	0.73 (0.25–2.13)	*0.57*	0.58 (0.18–1.88)	*0.37*
*Other*	23 (5)	2 (2)	0.32 (0.74–1.38)	*0.08*	0.27 (0.06–1.28)	*0.081*	0.27 (0.54–1.32)	*0.11*
Parity								
*Nulliparous*	143 (34)	47 (44)	Reference		Reference		Reference	
*Multiparous*	277 (66)	61 (56)	0.67 (0.44–1.03)	*0.07*	**0.58 (0.36–0.93)**	***0.03***	**0.56 (0.33–0.89)**	***0.02***
ART								
*None*	383 (91)	99 (92)	Reference		Reference		Reference	
*ART*	37 (9)	9 (8)	0.94 (0.44–2.01)	*0.88*	1.01 (0.44–2.29)	*0.99*	0.85 (0.35–2.04)	*0.71*
Smoking								
No	332 (79)	66 (61)	Reference		Reference		Reference	
Ex	69 (16)	27 (25)	**1.96 (1.17–3.30)**	***0.01***	**1.86 (1.08–3.24)**	***0.03***	**1.85 (1.05–3.25)**	***0.03***
Current	19 (5)	15 (14)	**3.97 (1.92–8.21)**	***< 0.001***	**4.24 (1.88–9.54)**	***< 0.001***	**4.22 (1.83–9.75)**	***0.001***
Housing								
Rented	123 (29)	45 (42)	Reference		Reference		Reference	
Owns	297 (71)	63 (58)	**0.58 (0.37–0.90)**	***0.01***	**0.57 (0.34–0.97)**	***0.04***	**0.56 (0.32–0.98)**	***0.04***
IMD Quintile								
*1st*	73 (18)	19 (18)	Reference		Reference		Reference	
*2nd*	78 (20)	29 (27)	1.42 (0.74–2.74)	*0.29*	1.31 (0.66–2.60)	*0.53*	1.37 (0.68–2.76)	*0.38*
*3rd*	97 (24)	13 (12)	0.51 (0.24–1.11)	*0.09*	0.46 (0.21–101)	*0.05*	0.48 (0.21–1.10)	*0.08*
*4th*	68 (17)	21 (20)	1.19 (0.59–2.40)	*0.63*	0.92 (0.44–1.93)	*0.82*	0.98 (0.46–2.10)	*0.97*
*5th*	82 (21) 22 missing values	14 (23) 2 missing values	1.12 (0.57–2.22)	*0.74*	0.98 (0.46–2.08)	*0.96*	1.02 (0.48–2.23)	*0.94*
Paternal Age								
*< 30 years*	86 (21)	33 (32)	Reference				Reference	
*30–34 years*	58 (14)	16 (15)	0.77 (0.39–1.51)	*0.45*			0.86	*0.72*
*35–39 years*	115 (28)	24 (23)	**0.54 (0.30–0.98)**	***0.04***			0.54	*0.16*
*≥40 years*	148 (36) 13 missing values	31 (90) 4 missing values	**0.54 (0.31–0.94)**	***0.03***			0.46	*0.08*

Model 1 – multivariable logistic regression including all variables except paternal age (ethnicity, parity, smoking, house ownership, IMD quintile and maternal age group), Model 2 – multivariable regression additionally adjusting for paternal age. *ART* Assistive Reproductive Techniques, *IMD* Index of multiple deprivation, *IMD* Quintile 1st = least deprived, 5th = most deprived. Statistics performed were univariate and multivariable meta-regression. Adjustments included maternal ethnicity, parity, smoking status, housing, and paternal age

### Nested case-control study to determine whether advanced maternal age associated with elevated circulating biomarkers of inflammation and oxidative stress

The characteristics of participants (*n* = 40/group) in this nested study are shown in Supplementary Table 2. The three groups differed for factors strongly associated with older age: higher home-ownership in 35–39 and ≥ 40 year olds (95% of ≥40 vs. 90% of 35–39 vs. 58% of 20–30 years) and multiparous (78% of ≥40 vs. 80% of 35–39 vs. 53% of 20–30 yeas) and mothers ≥40 years had higher previous miscarriage rates (53% vs. 25% of 20–30 years). Older mothers gave birth earlier (by 5–7 days) than 20–30 year olds (*p* < 0.001; Supplementary Table 3).

**Fig. 2:**
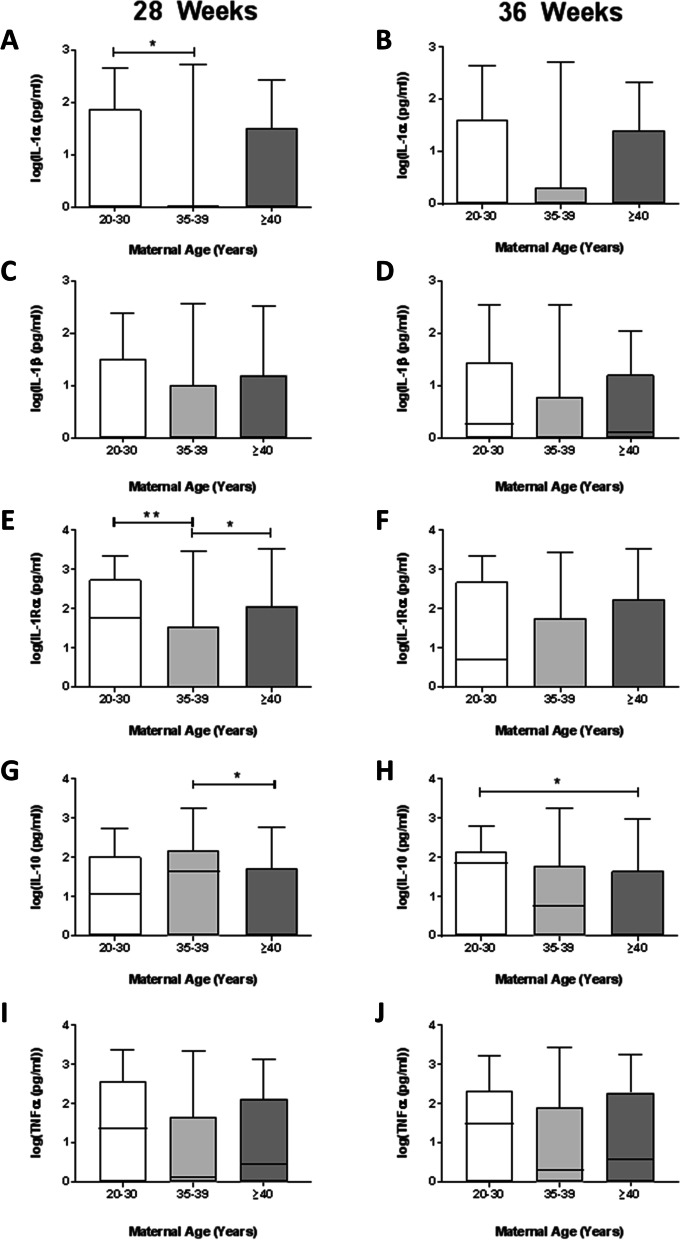
Circulating inflammatory markers in women grouped by maternal age, *n* = 40/group**.** Maternal serum at 28 weeks (**A**, **C**, **E**, **G**, **I**) and 36 weeks (**B**, **D**, **F**, **H**, **J**) gestation concentrations of: **A**, **B** interleukin (IL-1) α, **C**, **D** IL-1β, **E**, **F** IL-1Ra, **G**, **H** IL-10 and (I-J) TNFα. Data are logarithmically transformed, with median, interquartile range (box) and total range (whiskers) plotted. Analysed using one-way ANOVA (*p < 0.05, **p < 0.01)

**Fig. 3:**
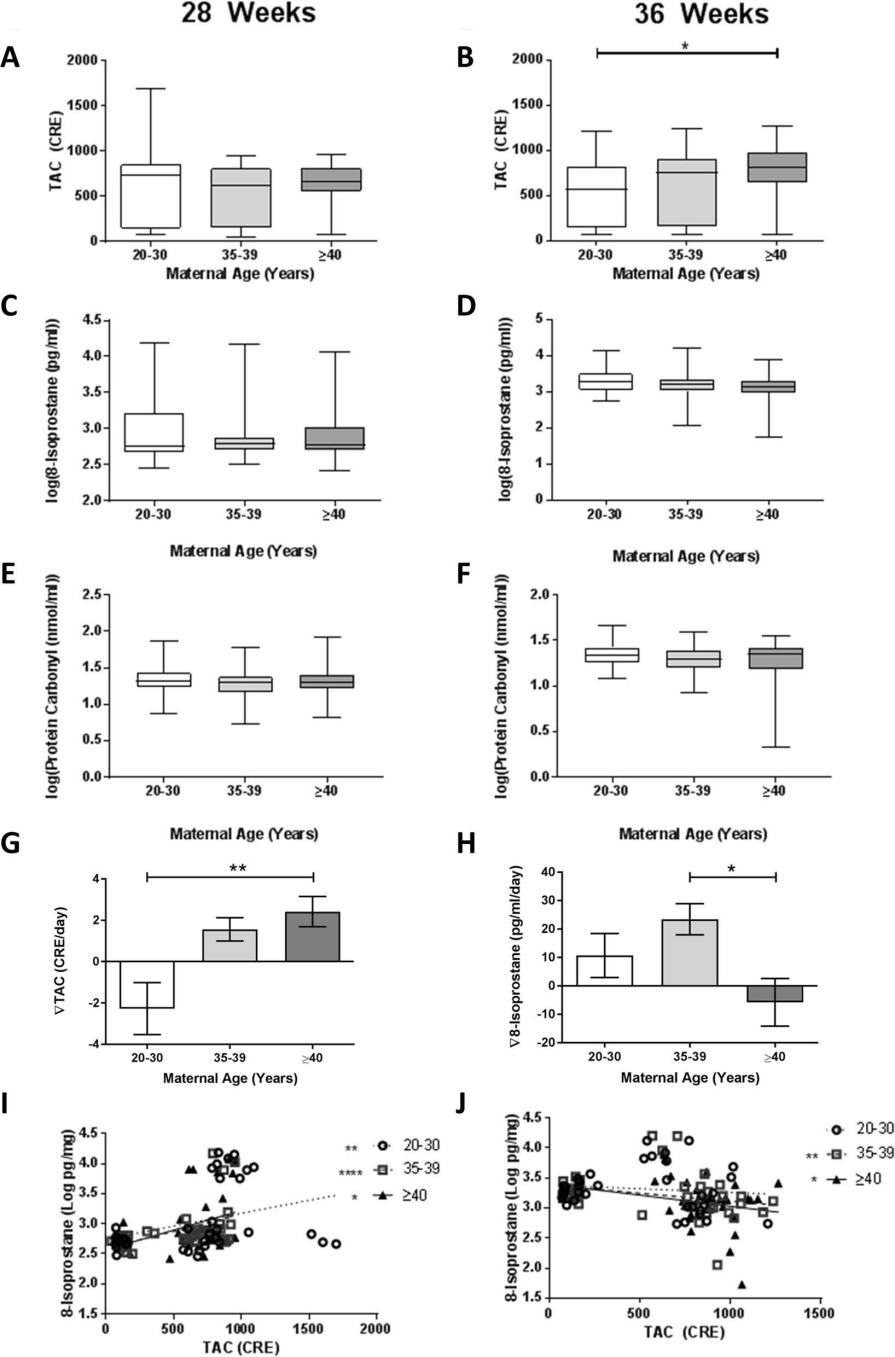
Levels of markers of oxidative stress status in women grouped by maternal age; *n* = 40/group**.** Maternal serum at 28 weeks (**A**, **C**, **E**) and 36 weeks (**B**, **D**, **F**) gestation was quantified for: **A**, **B** Total Antioxidant Capacity (TAC), **C**, **D** 8-Isoprostane and **E**, **F** protein Carbonyl. Rate of change between 28 and 36 weeks’ gestation of: **G** Total Antioxidant Capacity (TAC) and **H** 8-Isoprostane. Data presented as median, interquartile range (box) and total range (whiskers). Analysed using Kruskal-Wallis with Dunn’s multiple comparisons or one-way ANOVA on transformed data. TAC was correlated with log 8-isoprostane at **I** 28 weeks and **J** 36 weeks using Spearman rank test (*p < 0.05, **p < 0.01), *** *p* < 0.001)

Large variation was seen in inflammatory biomarker concentrations in maternal serum. At 28 weeks’ gestation, lower circulating concentrations of IL-1α and IL-1RA (*p* = 0.04 and 0.005, Fig. 2A, E) were measured in mothers 35–39 years compared to 20–30 years, but no significant differences were present between 20 and 30 and ≥ 40 year olds. Similar trends were apparent at 36 weeks gestation but were not statistically significant. Anti-inflammatory IL-10 was lower in mothers ≥40 years at 28 weeks’ (*p* = 0.05), with a stepwise age-related decrease apparent at 36 weeks (*p* = 0.03, Figs. 2G-H). No differences were seen in IL-1β or TNF-α at 28 weeks or 36 weeks between age groups (Fig. 2). There were no differences in maternal circulating markers of oxidative stress at 28 weeks’ gestation (Fig. 3A, C, E). TAC was increased at 36 weeks’ gestation in mothers ≥40 years compared to controls (*p* = 0.015; Fig. 3B). No differences of other oxidative stress markers were seen (Fig. 3D and F, Supplementary Figure 1). Gestational age affected TAC in mothers ≥40s (*p* = 0.004), whereas 8-isoprostane was positively related to gestational age in all groups (*p* < 0.001; data not shown). When assessing change in oxidative status over time, TAC levels fell across the third trimester in controls but increased in older women (*p* = 0.005; Fig. 3G). In contrast, elevated lipid peroxidation (8-isoprostane) was apparent in mothers 20–30 and 35–39 years across the third trimester but decreased in women ≥40 (*p* = 0.04; Fig. 3H). There was a positive relationship between TAC and 8-isoprostane in all participants at 28 weeks’ gestation, strongest in women 35–39 years (*r* = 0.61 vs 0.45 in women aged 20–30, Fig. 3I). At 36 weeks a negative correlation existed between TAC and 8-isoprostane in women 35–39 and ≥ 40 years (*r* = − 0.42 and − 0.39 respectively; *p* = 0.005 and *p* = 0.01 Fig. 3J), whereas no relationship was seen in women aged 20–30 (*r* = − 0.17, *p* = 0.32).

### Nested case-control study to determine whether maternal biomarkers associated with adverse pregnancy outcome in older women

Women ≥35 years of age who had an adverse pregnancy outcome (*n* = 43) were well matched for demographic variables to women ≥35 years of age who had a normal pregnancy outcome (Supplementary Table 4). The majority of infants in the group of women with adverse pregnancy outcome were classified as SGA (84%), with 44% under the 5th centile (FGR) (Supplementary Table 5). There were fewer NVDs (39% vs. 65%, *p* = 0.02) and more EMCS (23% vs. 7%, *p* = 0.04) in the group with adverse pregnancy outcomes compared to those with a normal outcome. A quarter of mothers in the adverse pregnancy outcome group delivered preterm (< 37 weeks, *p* < 0.001) and a quarter of infants were admitted to NICU (*p* < 0.001). There were three stillbirths included in the adverse pregnancy outcome group (one stillbirth was excluded due to congenital abnormality identified as the cause of death).

**Fig. 4:**
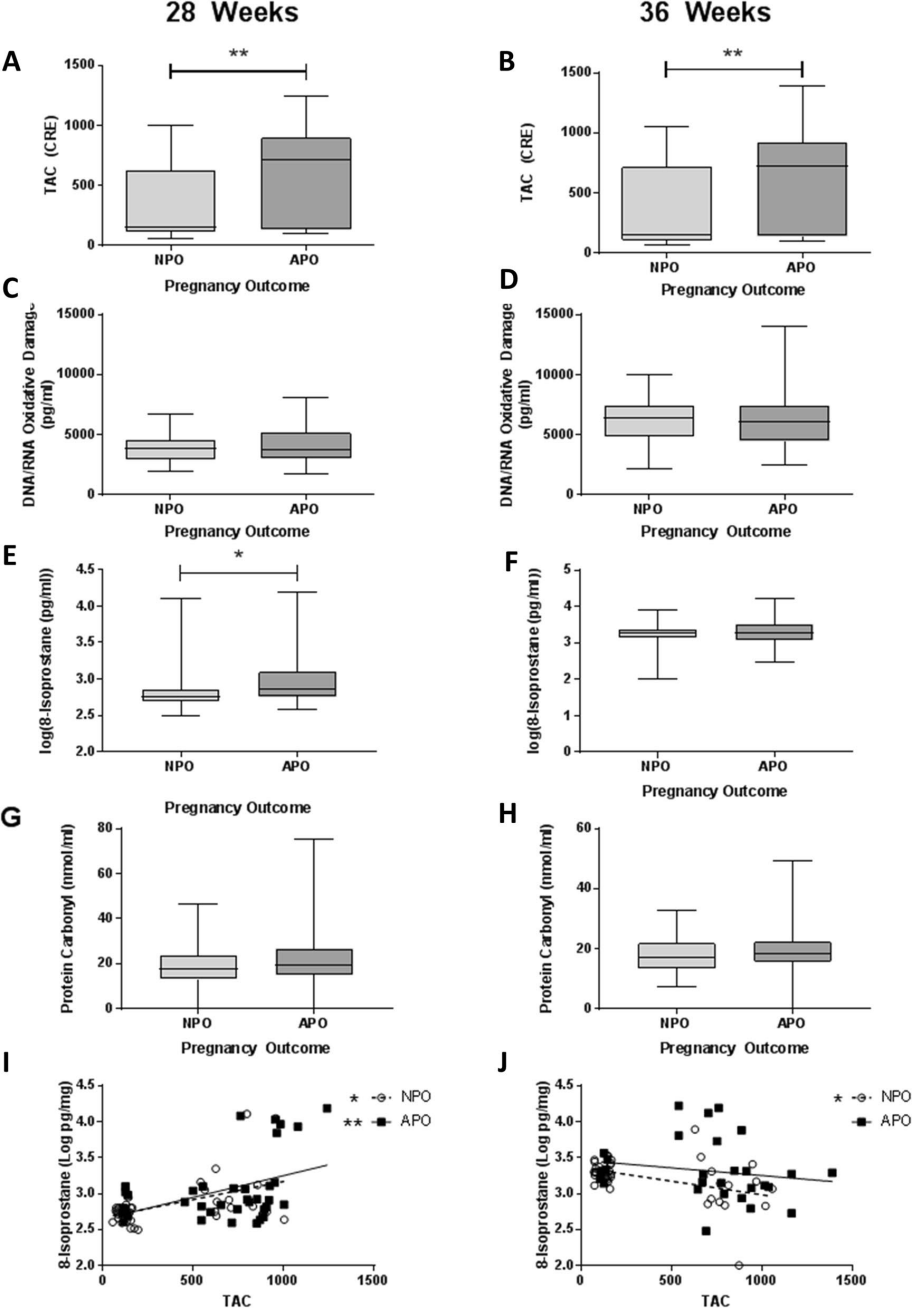
Levels of oxidative stress markers maternal serum from women ≥ 40 years of age with normal and adverse pregnancy outcome; *n* = 43/group. Maternal serum at 28 weeks (**A**, **C**, **E**, **G**) and 36 weeks (**B**, **D**, **F**, **H**) gestation against pregnancy outcome. **A**, **B** Total Antioxidant Capacity (TAC), **C**, **D** DNA/RNA damage, **E**, **F** 8-Isoprostane and **G**, **H** Protein Carbonyl. Data are (**A**, **B**) median, IQR and range, Mann Whitney test or (**C**-**H**) unpaired t-test for transformed data. TAC was correlated with 8-isoprostane at (**G**) 28 weeks and (**H**) 36 weeks gestation using Spearman rank test (*p < 0.05, **p < 0.01)

There were no differences in circulating cytokines at 28 weeks’ or 36 weeks’ gestation between older women with normal and adverse pregnancy outcome (Supplementary Figure 2). TAC was higher in older mothers with adverse pregnancy outcome compared to normal outcomes at both 28 and 36 weeks’ gestation (*p* = 0.002 and 0.006 respectively, Fig. 4A, B). 8-isoprostane increased between 28 and 36 weeks’ gestation in both normal and adverse pregnancy outcome groups (data not shown). 8-isoprostane also significantly increased in older mothers with adverse pregnancy outcome at 28 weeks but was not elevated at 36 weeks’ gestation (*p* = 0.01 and *p* = 0.82 respectively, Fig. 4E-F). No differences were detected in markers of DNA/RNA damage or protein carbonyl between groups at either 28 weeks (*p* = 0.66 and 0.34 respectively) or 36 weeks’ gestation (*p* = 0.57 and 0.60 respectively; Fig. 4C, D, G, H). A positive correlation between TAC and 8-isoprostane at 28 (*r* = 0.46 for adverse pregnancy outcome and 0.31 for normal pregnancy outcome) and at 36 weeks with normal outcomes (*r* = − 0.39; *p* = 0.01 Fig. 4J), whereas there was no relationship seen in women with adverse pregnancy outcome (*r* = − 0.33, *p* = 0.07, Fig. 4I-J).

**Fig. 5:**
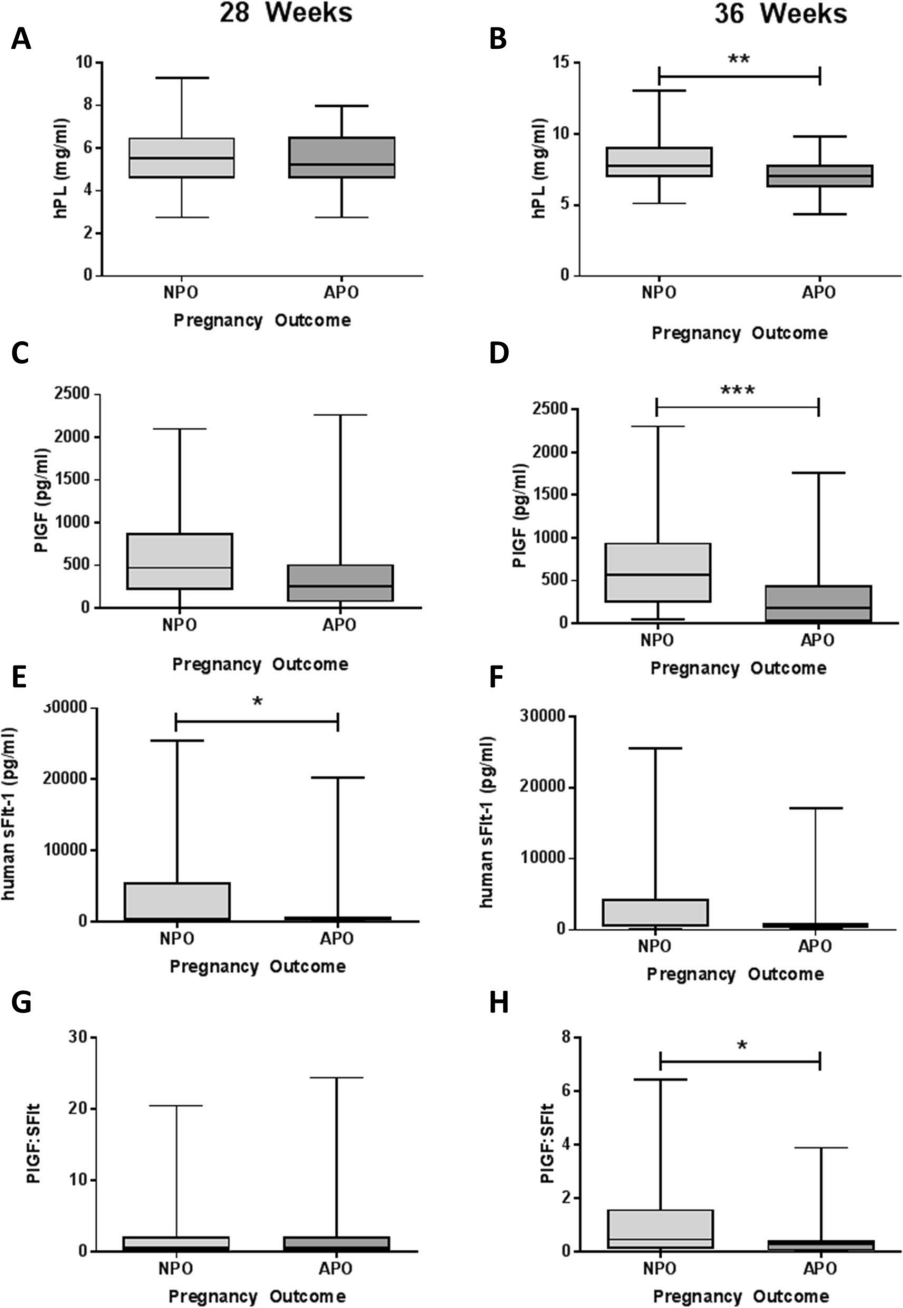
Levels of placental hormones in maternal serum from women ≥ 40 years of age with normal and adverse pregnancy outcome (*n* = 43/group). Maternal serum/plasma at 28 weeks (**A**, **C**, **E**, **G**) and 36 weeks (**B**, **D**, **F**, **H**) gestation against pregnancy outcome. **A**, **B** human placental lactogen (hPL), **C**, **D** Placental growth factor (PlGF), **E**, **F** Soluble fms like tyrosine kinase-1 (sFlt) and **G**, **H** PlFG:sFlt ratio. Data presented as median, interquartile range (box) and total range (whiskers) and analysed using Mann-Whitney U-test (*p < 0.05, **p < 0.01, ***p < 0.005)

**Fig. 6:**
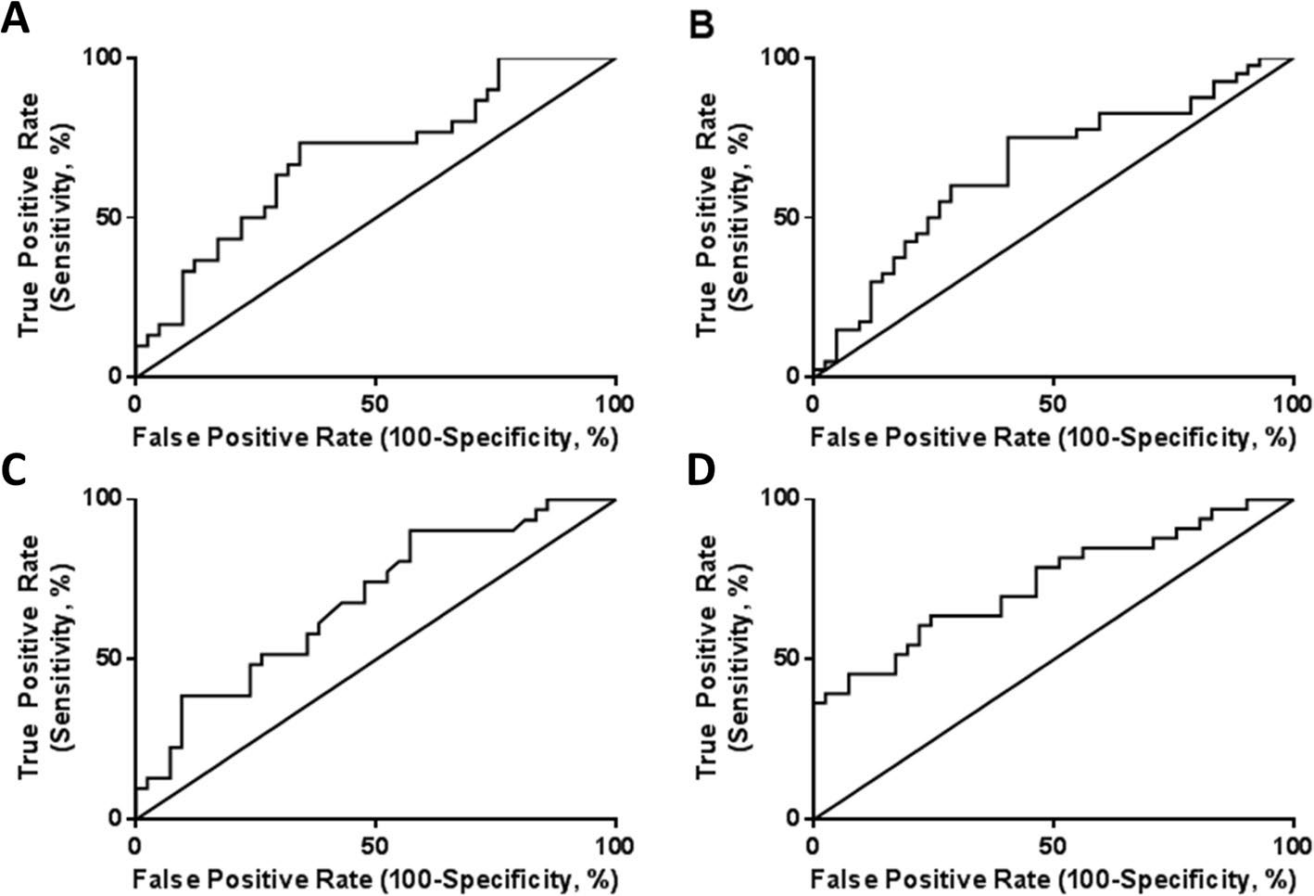
Predictive values of biomarkers of adverse pregnancy outcome in women of advanced maternal age. ROC curve of (**A**) TAC (Area under the ROC curve (AUROC) =0.69), (**B**) 8-Isoprostane (AUROC = 0.66), (**C**) hPL (AUROC = 0.68) and (**D**) PlGF (AUROC = 0.74) as predictors of adverse pregnancy outcome

Concentrations of hPL, PlGF and PlGF:sFlt ratio were unchanged at 28 weeks’ gestation (Fig. 5A, C, G). hPL was lower at 36 weeks’ gestation in women with adverse pregnancy outcome (*p* = 0.007; Fig. 5B). Similarly, PlGF concentrations were lower at 36 weeks’ when measured alone (*p* < 0.001, Fig. 5D) or adjusted for sFlt-1 (human VEGF R1/Flt-1) concentrations (*p* = 0.03, Fig. 5H). sFlt-1 was lower in women with adverse pregnancy outcome at 28 weeks’ with a similar trend at 36 weeks’ gestation (*p* = 0.05 and 0.07, Fig. 5E,F). No differences were detected in circulating hCG, PAPP-A or progesterone with adverse pregnancy outcome (Supplementary Figure 3). ROC curves were created for all biomarkers that reached a statistical significance of *p* < 0.01 between normal pregnancies and those with adverse pregnancy outcome. TAC and 8-isoprostane had predictive area under the curve values of 0.69 and 0.66 respectively (ranked as a poor prognostic markers, Fig. 6A-B), whilst hPL and PlGF had predictive values of 0.68 (poor) and 0.74 (fair), respectively (Fig. 6C-D).

## Discussion

This prospective study found that increased maternal age was associated with increased total antioxidant capacity and a reduction in anti-inflammatory IL-10 and IL-RA indicating changes in oxidative stress and inflammation over the timeframe of reproductive life span. In mothers ≥35 years of age, adverse pregnancy outcome was associated with a further increase in total antioxidant capacity and a reduction in placentally-derived biomarkers, hPL and PlGF. In this population, maternal smoking and primiparity were independent risk factors for adverse outcome, consistent with retrospective study findings [7, 10, 38].

### Strengths and limitations

This prospective study was designed to explore potential mechanisms underpinning the association between advanced maternal age and adverse pregnancy outcome, to identify potential biomarkers and generate further hypotheses which can be explored. The multi-centre approach used in this study offered diversity, making it more representative of the UK maternity population. However, this study would have benefitted from larger overall sample size to increase the statistical power to confirm associations between adverse pregnancy outcome and biomarkers and assess the predictive ability of combinations of clinical and biochemical markers. Furthermore, a larger cohort would have enabled larger nested case control studies, with greater statistical power, particularly for cytokine biomarkers which had wide variation in levels between individuals. In addition, the study excluded women with a BMI above 30, which may account for some of the increased adverse outcomes observed in older women (as older women have a higher BMI than younger women). We recognize this limitation of the study and believe that BMI is an important covariate to include in future studies to determine the degree of interaction between these two variables that are independently associated with adverse pregnancy outcome.

Despite these limitations, MAMAS is a large prospective study investigating associated factors for adverse pregnancy outcome in older women and our detailed data collection provided the ability to adjust for multiple confounding variables, and delineate the effects of paternal age, ethnicity and parity and socioeconomic status, all of which may be associated with stillbirth [39–42].

### Interpretation

Consistent with other larger retrospective studies the clinical factors associated with adverse outcome in older women were maternal smoking and primiparity [8, 10, 38]. However, in our study population conception by ART had no significant association with stillbirth, although the effect-size of other studies was within the 95% confidence intervals of our population [43, 44]. It is notable that in this and other studies, older mothers were more likely to own their own homes, be in a stable relationship, have higher rates of education and had the lowest rates of cigarette smoking [45], factors which are associated with lower risk of adverse outcomes such as stillbirth and fetal growth restriction [46]. However, the opposite finding that older women have higher rates of these conditions is noted in observational studies [8]. These findings suggest that other mechanisms much account for the increased rate of adverse maternal and neonatal outcomes in older mothers. Furthermore, they also emphasise the need to promote smoking cessation services and suggest that women who have their first pregnancy over the age of 35 or those who smoke should be prioritised for intervention.

Understanding the mechanisms underlying the susceptibility to adverse outcomes is essential to improve identification of older women highest risk of adverse outcomes. This study focussed on processes implicated in maternal aging and placental dysfunction. A pro-inflammatory bias and elevated oxidative stress are established hallmarks of aging [47–49] and both are strongly associated with pregnancy pathologies, particularly those characterised by placental dysfunction [50–52]. The nested case control studies revealed features of biological aging in older women in the absence of adverse outcome including: elevations in TAC, coincident with reduced oxidative damage (reduced lipid peroxidation) and a reduction in anti-inflammatory cytokines (IL-10 and IL-1Ra) possibly indicating a shift towards a pro-inflammatory state. The former findings are consistent with these women delivering healthy infants and suggest that adaptive antioxidant responses are effective in maintaining the oxidant:antioxidant balance protecting against oxidative stress [53]. The reduction in IL-10 levels has also been seen in serum and placenta of women perceiving reduced fetal movements [27] and in the placenta of infants with FGR [54]. As both FGR and RFM are associated with placental dysfunction, the reduction in IL-10 could be consistent with the increased placental dysfunction seen in older mothers [28]. Critically, an isolated reduction in anti-inflammatory status is not detrimental, but studies of the IL-10 knockout mouse demonstrate increased susceptibility to inflammatory stimuli resulting in PTB and fetal loss [55], and exacerbation of the vascular symptoms of preeclampsia [56] and effects of hypoxia [57]. Therefore, an age-related decline in anti-inflammatory cytokines may increase vulnerability of older women to inflammation and the associated detrimental effects observed on placental function and should therefore be further explored [58]. We speculate that maternal aging creates a suboptimal environment for placental and fetal development that contributes to the vulnerability to adverse outcomes.

In this population adverse pregnancy outcome was associated with higher circulating levels of 8-isoprostane at 28 weeks’ gestation, indicating elevated oxidative damage, despite higher antioxidant capacity. Inadequate antioxidant compensatory responses resulting in oxidative stress has been detected in placentas from adverse pregnancy outcome, where it has been related to altered placental function [22]. Future studies are required to confirm whether placental oxidative damage is implicated in the placental dysfunction observed in pregnancies in older mothers.

Consistent with other reports reduced maternal circulating concentrations of placental hormones (hPL, sFlt and PlGF) were detected in pregnancies in older women with adverse outcome compared to normal outcomes. The differences in placental hormones provide further evidence for placental dysfunction as an underpinning mechanism for susceptibility to adverse outcomes in older women. There is a growing body of evidence that these represent biomarkers of placental dysfunction, and are lower in pregnancies with FGR, PE and stillbirth [59–61]. PlGF and sFlt concentrations in the maternal circulation are correlated with fetal size as early as the first trimester [62] and have strong potential as biomarkers for adverse pregnancy outcome in a clinical setting [61, 63]. In common with prognostic accuracy studies from other contexts in pregnancy, PlGF had the strongest predictive value for adverse pregnancy outcome in older mothers [64], although the AUROC is insufficient for clinical utility at present. Further studies with larger sample size are required to determine if a prognostic model incorporating the clinical and biochemical predictors with sufficient predictive power to identify older women at greatest risk of adverse outcome can be derived.

## Conclusion

This study has identified alterations in circulating biomarkers of inflammation and oxidative stress markers in older pregnant women, suggesting that biological processes seen in aging may contribute to susceptibility to adverse outcomes in this population. Furthermore, we identified serum biomarkers with fair predictive accuracy for adverse pregnancy outcome in older women. With larger sample sizes and data sets, and by combining identified demographic and clinical variables that alter risk of adverse outcome and the measurement of aging and placental biomarkers, it may be possible to create a predictive model with sufficient discrimination to delineate an individual woman’s risk of adverse pregnancy outcome relating to advanced maternal age. This would allow older mothers to be offered more individualised care that considers both maternal and fetal wellbeing and reduces stillbirth rates whilst minimising unnecessary intervention.

## Supplementary Information

**Additional file 1.**

## Data Availability

The datasets generated and/or analysed during the current study are not publicly available as ethnical approval was not sought for their dissemination but are available from the corresponding author on reasonable request.

## Abbreviations

AOR: Adjusted odds ratio
ART: Assisted reproductive technology
AUROC: Area under the receiver operator characteristic curve
BMI: Body mass index
ELISA: Enzyme linked immunosorbent assay
FGR: Fetal growth restriction
hPL: Human placental lactogen
IBC: Individualised birthweight centile
IL: Interleukin
IMD: Index of multiple deprivation
IOL: Induction of labour
MAMAS: Manchester Advanced Maternal Age Study
NICU: Neonatal intensive care unit
OR: Odds ratio
PE: Preeclampsia
PlGF: Placental growth factor
PTB: Preterm birth
sFlt1: Soluble fms-like tyrosine kinase 1
TNF: Tumour necrosis factor
