# Supplementary material for: A prospective cohort study providing insights for markers of adverse pregnancy outcome in older mothers
Source: BMC Pregnancy Childbirth. 2021 Oct 20;21:706. doi: 10.1186/s12884-021-04178-6 (PMC8527686; doi:10.1186/s12884-021-04178-6)
Supplement: Supplementary file 1 — Additional file 1. [file 12884_2021_4178_MOESM1_ESM.zip › Supplementary Figure 2.docx]

# **A Prospective Cohort Study providing Insights for Markers of Adverse Pregnancy Outcome in Women of Advanced Maternal Age**

Samantha C. LEAN, Maternal and Fetal Health Research Centre, Division of Developmental Biology and Medicine, Faculty of Biology, Medicine and Health, University of Manchester, UK. sl961@cam.ac.uk

Rebecca L. JONES, Maternal and Fetal Health Research Centre, Division of Developmental Biology and Medicine, Faculty of Biology, Medicine and Health, University of Manchester, UK. rebecca.lee.jones@manchester.ac.uk

Stephen A. ROBERTS, Centre for Biostatistics, Faculty of Biology, Medicine and Health, University of Manchester, UK. steve.roberts@manchester.ac.uk

Alexander E.P. HEAZELL, Maternal and Fetal Health Research Centre, Division of Developmental Biology and Medicine, Faculty of Biology, Medicine and Health, University of Manchester, UK


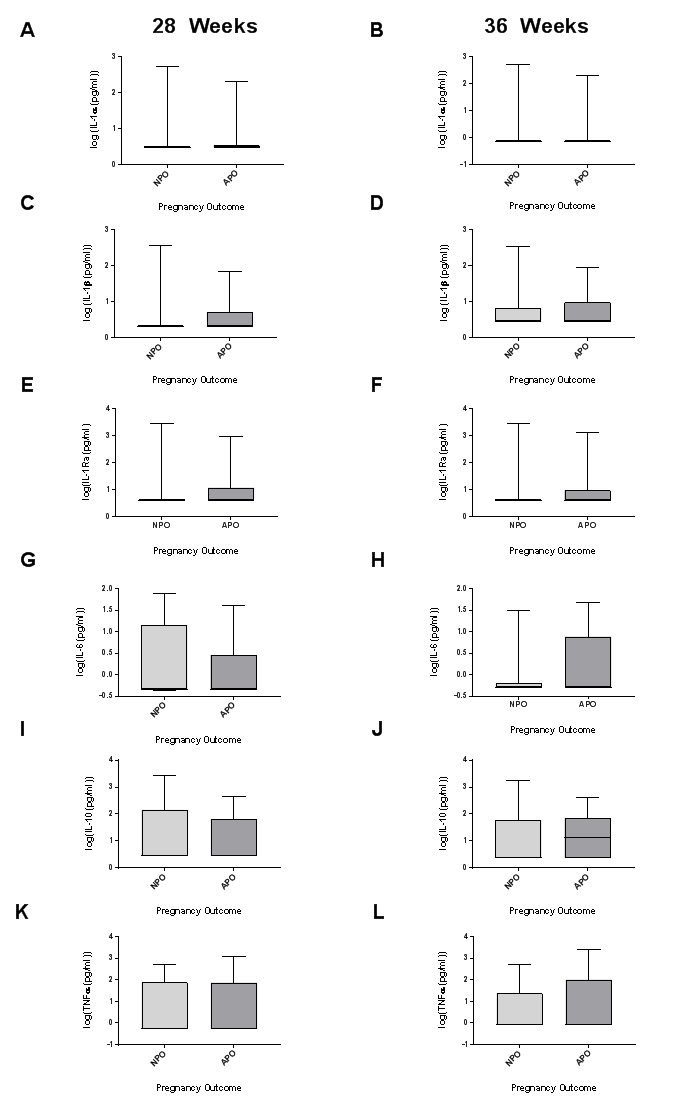


**Supplementary Figure 2: Circulating inflammatory markers in women of advanced maternal age with normal and adverse pregnancy outcome,** n=43/group. Maternal serum at 28 weeks (A, C, E, G, I, K) and 36 weeks (B, D, F, H, J, L) gestation were quantified for: (A-B) interleukin (IL)-1α (C-D) IL-1β (E-F) IL-1Ra (G-H) IL-6 (I-J) IL-10 and (K-L) TNFα. Data are logarithmic transformed mean ± SEM, unpaired t-test.
