# Supplementary material for: A prospective cohort study providing insights for markers of adverse pregnancy outcome in older mothers
Source: BMC Pregnancy Childbirth. 2021 Oct 20;21:706. doi: 10.1186/s12884-021-04178-6 (PMC8527686; doi:10.1186/s12884-021-04178-6)
Supplement: Supplementary file 1 — Additional file 1. [file 12884_2021_4178_MOESM1_ESM.zip › Supplementary Table 1.docx]

# **A Prospective Cohort Study providing Insights for Markers of Adverse Pregnancy Outcome in Women of Advanced Maternal Age**

Samantha C. LEAN, Maternal and Fetal Health Research Centre, Division of Developmental Biology and Medicine, Faculty of Biology, Medicine and Health, University of Manchester, UK. sl961@cam.ac.uk

Rebecca L. JONES, Maternal and Fetal Health Research Centre, Division of Developmental Biology and Medicine, Faculty of Biology, Medicine and Health, University of Manchester, UK. rebecca.lee.jones@manchester.ac.uk

Stephen A. ROBERTS, Centre for Biostatistics, Faculty of Biology, Medicine and Health, University of Manchester, UK. steve.roberts@manchester.ac.uk

Alexander E.P. HEAZELL, Maternal and Fetal Health Research Centre, Division of Developmental Biology and Medicine, Faculty of Biology, Medicine and Health, University of Manchester, UK

Supplementary Table 1: Aging biomarkers assay kits details with sample type, dilution factor and intra and inter-plate variability (%CV)

| **Assay** | **Sample** | **Dilution** | **Intra-Assay Variability** (%CV) | **Inter-Plate Variability**  (%CV) |
| --- | --- | --- | --- | --- |
| **Inflammatory Cytokines** | | | | |
| **IL-1α R&D DuoSet** | Serum | Undiluted | 6.7 | 1.2 |
| **IL-1β R&D DuoSet** | Serum | Undiluted | 6.0 | 14.0 |
| **IL-1Ra R&D DuoSet** | Serum | Undiluted | 3.9 | 11.0 |
| **IL-6 R&D DuoSet** | Serum | Undiluted | 9.9 | 2.9 |
| **IL-10 R&D DuoSet** | Serum | Undiluted | 15.7 | 12.0 |
| **TNFα R&D DuoSet** | Serum | Undiluted | 10.7 | 2.2 |
| **Oxidative Stress Markers** | | | | |
| **OxiSelect™ Total Antioxidant Capacity Assay Kit** | Plasma | Undiluted | 5.1 | 32.8 |
| **8-Isoprostane EIA Kit** | Plasma | Undiluted | 3.9 | 15.6 |
| **DNA/RNA Oxidative Damage EIA Kit Damage** | Plasma | Undiluted | 4.2 | 15.9 |
| **Protein Carbonyl Calorimetric Assay Kit** | Plasma | Undiluted | 8.5 | 19.0 |
| **Placental Hormones** | | | | |
| **PAPP-A DRG ELISA Kit** | Serum | 1:25 | 4.2 | 17.1 |
| **hCG DRG ELISA Kit** | Serum | Undiluted | 3.7 | 20.3 |
| **hPL DRG ELISA Kit** | Serum | 1:100 | 3.8 | 6.3 |
| **Progesterone DRG ELISA Kit** | Serum | 1:100 | 3.7 | 21.4 |
| **PlGF R&D DuoSet** | Plasma | 1:4 | 3.9 | 11.1 |
| **sFlt R&D DuoSet** | Plasma | 1:10 | 4.5 | 13.8 |

*%CV= percentage critical variance; PlGF = Placental Growth Factor; sFlt = soluble fms-like tyrosine kinase; hPL = human placental lactogen; PAPP-A= pregnancy associated plasma protein-A; hCG = human chorionic gonadotropin;*
