# Supplementary material for: A prospective cohort study providing insights for markers of adverse pregnancy outcome in older mothers
Source: BMC Pregnancy Childbirth. 2021 Oct 20;21:706. doi: 10.1186/s12884-021-04178-6 (PMC8527686; doi:10.1186/s12884-021-04178-6)
Supplement: Supplementary file 1 — Additional file 1. [file 12884_2021_4178_MOESM1_ESM.zip › Supplementary Table 2.docx]

# **A Prospective Cohort Study providing Insights for Markers of Adverse Pregnancy Outcome in Women of Advanced Maternal Age**

Samantha C. LEAN, Maternal and Fetal Health Research Centre, Division of Developmental Biology and Medicine, Faculty of Biology, Medicine and Health, University of Manchester, UK. sl961@cam.ac.uk

Rebecca L. JONES, Maternal and Fetal Health Research Centre, Division of Developmental Biology and Medicine, Faculty of Biology, Medicine and Health, University of Manchester, UK. rebecca.lee.jones@manchester.ac.uk

Stephen A. ROBERTS, Centre for Biostatistics, Faculty of Biology, Medicine and Health, University of Manchester, UK. steve.roberts@manchester.ac.uk

Alexander E.P. HEAZELL, Maternal and Fetal Health Research Centre, Division of Developmental Biology and Medicine, Faculty of Biology, Medicine and Health, University of Manchester, UK

Supplementary Table 2: Demographic data of participants in nested case cohort study 1 (NCC1)

| **Demographic** | **20-30 Years**  **(n=40)** | **35-39 Years**  **(n=40)** | **≥40 Years**  **(n=40)** | ***p* value**  **Overall** | ***p* value**  **Multiple Comparisons** |
| --- | --- | --- | --- | --- | --- |
| **Maternal Age**  (years) | **26** (20-30) | **37** (35-39) | **42** (40 – 48) | --- | --- |
| **Paternal Age ^a^**  (years) | **29** (21-39) | **36** (27-45) | **43** (25-55) | **<0.0001** | ^†¶^**0.0001,**  ^Δ^ **0.002** |
| **Ethnicity ^b^**  *European* | **98% (**39) | **98%** (39) | **98%** (39) | >0.99 | --- |
| **BMI ^a^**  (kg/m^2^) | **23.6**  (19.0-29.9) | **24.0**  (18.5 – 28.6) | **24.6**  (19.4-24.6) | >0.99 | --- |
| **Marital Status ^b^**  *Married*  *Partner*  *Single* | **50%** (20)  **48%** (19)  **3%** (1) | **70%** (28)  **30%** (12)  **0%** (0) | **70%** (28)  **25%** (10)  **5%** (2) | 0.104  0.79  0.23 | --- |
| **Employment ^b^**  *Employed* | **90%** (36) | **78%** (31) | **78%** (31) | 0.27 | --- |
| **Smoking ^b^**  *Non-Smokers* | **100%** (40) | **100%** (40) | **100%** (40) | >0.999 | --- |
| **Housing ^b^**  *Home owner* | **58%** (23) | **90%** (36) | **95**% (37) | **0.00016** | ^†^**0.0018,** ^¶^**0.0005,** ^Δ^1.0 |
| **IMD**  ***Score*** | **13.28**  (2.62-66.66) | **11.04**  (2.17-66.1) | **13.23**  (1.94-46.71) | 0.36 | **---** |
| **Parity ^b^**  *Primiparous*  *Parous*  *Grandmultiparous* | **48%** (19)  **53%** (21)  **0.0%** (0) | **20%** (8)  **80%**(32)  **3%** (1) | **23%** (9)  **78%** (31)  **8%** (3) | **0.035** | **---**  ^†^**0.017,** ^¶^**0.034,** ^Δ^1.0  **---** |
| **Previous Miscarriage ^b^** | **25%** (10) | **35%** (14) | **53%** (21) | **0.035** | ^†^0.46**,** ^¶^**0.021,** ^Δ^0.18 |
| **Previous APO ^b^**  *of parous women* | **14%** (3) | **24%** (8) | **24%** (8) | 0.25 | --- |
| **Fertility Treatment ^b^** | **0%** (0) | **0%** (0) | **0%** (0) | >0.99 | --- |

*Data are mean (range) or percentage (number). NCC1 (Young vs AMA with normal pregnancy outcomes); n=40/group. AMA = advanced maternal age; NPO = normal pregnancy outcome; BMI = body mass index; APO = adverse pregnancy outcome; Fertility Treatment = in current or previous pregnancies. Statistical differences are from control (20-30 years) group.* Statistical differences are ^a^Kruskal-Wallis with Dunn’s multiple comparisons or ^b^ Fishers Exact test. When overall *p*>0.05, multiple comparisons *p* values are reported (^†^ 20-30 vs. 35-39 years, ^¶^ 20-30 vs ≥40 years, ^Δ^ 35-39 vs. ≥40 years). Significant differences are highlighted with **bold** p values.
