# Supplementary material for: A prospective cohort study providing insights for markers of adverse pregnancy outcome in older mothers
Source: BMC Pregnancy Childbirth. 2021 Oct 20;21:706. doi: 10.1186/s12884-021-04178-6 (PMC8527686; doi:10.1186/s12884-021-04178-6)
Supplement: Supplementary file 1 — Additional file 1. [file 12884_2021_4178_MOESM1_ESM.zip › Supplementary Table 3.docx]

# **A Prospective Cohort Study providing Insights for Markers of Adverse Pregnancy Outcome in Women of Advanced Maternal Age**

Samantha C. LEAN, Maternal and Fetal Health Research Centre, Division of Developmental Biology and Medicine, Faculty of Biology, Medicine and Health, University of Manchester, UK. sl961@cam.ac.uk

Rebecca L. JONES, Maternal and Fetal Health Research Centre, Division of Developmental Biology and Medicine, Faculty of Biology, Medicine and Health, University of Manchester, UK. rebecca.lee.jones@manchester.ac.uk

Stephen A. ROBERTS, Centre for Biostatistics, Faculty of Biology, Medicine and Health, University of Manchester, UK. steve.roberts@manchester.ac.uk

Alexander E.P. HEAZELL, Maternal and Fetal Health Research Centre, Division of Developmental Biology and Medicine, Faculty of Biology, Medicine and Health, University of Manchester, UK

Supplementary Table 3: Obstetric outcomes of participants in nested case cohort study 1 (NCC1)

| **Demographic** | **20-30 Years**  **(n=40)** | **35-39 Years**  **(n=40)** | **≥40 Years**  **(n=40)** | ***p* value**  **Overall** | ***p* value**  **Multiple Comparisons** |
| --- | --- | --- | --- | --- | --- |
| **Gestation at Delivery ^a^**  (weeks + days) | **40+2**  (37+6 – 42+2) | **39+5**  (37+4- 42+1) | **39+2**  (37+1 – 42+4) | **0.0007** | ^†^0.39**,** ^¶^**0.0005,** ^Δ^0.60 |
| **Birthweight ^a^**  (g) | **3545**  (2965-4300) | **3375**  (2640 – 3920) | **3401**  (2800 – 4250) | 0.11 | --- |
| **IBC ^a^** | **46.4**  (11.9-94.4) | **40.1**  (10.4-76.0) | **46.9**  (11.1 – 89.1) | 0.44 | --- |
| **Induction ^b^** | **27.5%** (11) | **17.5%** (7) | **37.5%** (15) | 0.13 | --- |
| **Mode of Delivery ^b^**  *NVD*  *ELCS*  *EMCS*  *INST.* | **53%** (21)  **18%** (7)  **8%** (3)  **23%** (9) | **65%** (26)  **23%** (9)  **5%** (2)  **8%** (3) | **58%** (23)  **18%** (7)  **15%** (6)  **10%** (4) | 0.84 | ---  ---  ---  --- |
| **Male Infant ^b^** | **55%** (22) | **40%** (16) | **55%** (22) | 0.32 | --- |
| **Composite APO ^b^** | **0%** (0) | **0%** (0) | **0%** (0) | >0.99 | --- |

Data are mean (range) or percentage (number). NCC1 (Young vs AMA mothers with normal pregnancy outcomes); n=40/group. IBC = individualised birthweight centile, NVD = normal vaginal delivery, ELCS = elective caesarean section, EMCS= emergency caesarean section, INST. = instrumental delivery. Statistics are ^a^Kruskal-Wallis with Dunn’s multiple comparisons or ^b^ Fishers Exact test. When overall *p*>0.05, multiple comparisons *p* values are reported (^†^ 20-30 vs. 35-39 years, ^¶^ 20-30 vs ≥40 years, ^Δ^ 35-39 vs. ≥40 years. Significant differences are highlighted with **bold** p values
