# Supplementary material for: A prospective cohort study providing insights for markers of adverse pregnancy outcome in older mothers
Source: BMC Pregnancy Childbirth. 2021 Oct 20;21:706. doi: 10.1186/s12884-021-04178-6 (PMC8527686; doi:10.1186/s12884-021-04178-6)
Supplement: Supplementary file 1 — Additional file 1. [file 12884_2021_4178_MOESM1_ESM.zip › Supplementary Table 4.docx]

# **A Prospective Cohort Study providing Insights for Markers of Adverse Pregnancy Outcome in Women of Advanced Maternal Age**

Samantha C. LEAN, Maternal and Fetal Health Research Centre, Division of Developmental Biology and Medicine, Faculty of Biology, Medicine and Health, University of Manchester, UK. sl961@cam.ac.uk

Rebecca L. JONES, Maternal and Fetal Health Research Centre, Division of Developmental Biology and Medicine, Faculty of Biology, Medicine and Health, University of Manchester, UK. rebecca.lee.jones@manchester.ac.uk

Stephen A. ROBERTS, Centre for Biostatistics, Faculty of Biology, Medicine and Health, University of Manchester, UK. steve.roberts@manchester.ac.uk

Alexander E.P. HEAZELL, Maternal and Fetal Health Research Centre, Division of Developmental Biology and Medicine, Faculty of Biology, Medicine and Health, University of Manchester, UK

Supplementary Table 4: Demographic data of participants in nested case cohort study 2 (NCC2)

| **Demographic** | **NPO**  **(n=43)** | **APO**  **(n=43)** | ***p* value** |
| --- | --- | --- | --- |
| **Maternal Age ^a^**  (years) | **39** (35-47) | **39** (35-46) | 0.902 |
| **Paternal Age ^a^**  (years) | **39** (27-56) | **40** (25-60) | 0.99 |
| **Ethnicity ^b^**  *European*  *Other* | **88%** (38)  **12%** (5) | **91%** (39)  **9%** (4) | 0.74 |
| **BMI** (kg/m^2^) **^a^** | **29.0** (18.5-24.3) | **25.0** (20-29.1) | 0.52 |
| **Marital Status ^b^**  *Married*  *Partner*  *Single* | **61%** (26)  **37%** (16)  **2%** (1) | **40%** (17)  **58%** (25)  **2%** (1) | 0.057 |
| **Employment ^b^**  *Employed* | **74%** (32) | **84%** (36) | 0.43 |
| **Smoking Status ^b^**  *Smoker*  *Ex-smoker*  *Non-smoker* | **12%** (5)  **16%** (7)  **72%** (31) | **19%** (8)  **30%** (13)  **51%** (22) | 0.051 |
| **Housing Status ^b^**  *Owns* | **77%** (33) | **67%** (29) | 0.35 |
| **IMD**  *Score* | **20.13**  (3.32-66.1) | **13.5**  (2.16-53.4) | 0.11 |
| **Parity ^b^**  *Primips*  *Parous*  *Grandmultiparous* | **28%** (12)  **72%** (31)  **7%** (3) | **44%** (19)  **56%** (23)  **2%** (1) | 0.081  0.20  0.37 |
| **Previous Miscarriage ^b^** | **42%** (18) | **44%** (19) | >0.99 |
| **Previous APO ^b^**  *(of parous women)* | **29%** (9) | **46%** (11) | 0.25 |
| **Previous Fertility Treatment ^b^** | **7%** (3) | **2%** (1) | 0.37 |
| **Current Fertility Treatment ^b^** | **7%** (3) | **7%** (3) | 1.0 |

*NCC2 (**AMA normal pregnancy outcome (NPO) vs adverse pregnancy outcome (APO)); n=43/group. Data are mean (range) or percentage (number). BMI = body mass index, APO = adverse pregnancy outcome. ^a^Mann-Whitney U Test or ^b^Fisher’s exact test.* Significant differences are highlighted with **bold** p values.
