# Supplementary material for: A prospective cohort study providing insights for markers of adverse pregnancy outcome in older mothers
Source: BMC Pregnancy Childbirth. 2021 Oct 20;21:706. doi: 10.1186/s12884-021-04178-6 (PMC8527686; doi:10.1186/s12884-021-04178-6)
Supplement: Supplementary file 1 — Additional file 1. [file 12884_2021_4178_MOESM1_ESM.zip › Supplementary Table 5.docx]

# **A Prospective Cohort Study providing Insights for Markers of Adverse Pregnancy Outcome in Women of Advanced Maternal Age**

Samantha C. LEAN, Maternal and Fetal Health Research Centre, Division of Developmental Biology and Medicine, Faculty of Biology, Medicine and Health, University of Manchester, UK. sl961@cam.ac.uk

Rebecca L. JONES, Maternal and Fetal Health Research Centre, Division of Developmental Biology and Medicine, Faculty of Biology, Medicine and Health, University of Manchester, UK. rebecca.lee.jones@manchester.ac.uk

Stephen A. ROBERTS, Centre for Biostatistics, Faculty of Biology, Medicine and Health, University of Manchester, UK. steve.roberts@manchester.ac.uk

Alexander E.P. HEAZELL, Maternal and Fetal Health Research Centre, Division of Developmental Biology and Medicine, Faculty of Biology, Medicine and Health, University of Manchester, UK

**Supplementary Table 5: Obstetric outcomes of participants in nested case cohort study 2 (NCC2)**

| **Demographics** | **NPO**  **(n=43)** | **APO**  **(n=43)** | ***p* value** |
| --- | --- | --- | --- |
| **Gestation at Delivery ^a^**  (weeks + days) | **39+5** (37+6 – 42+3) | **38+3** (29+6 - 41+3) | **0.007** |
| **Birthweight** (g) **^a^** | **3439** (2960-4000) | **2537** (1300-3690) | **<0.0001** |
| **IBC ^a^** | **45.1** (13.0-92.2) | **10.8** (0.0-91.4) | **<0.0001** |
| **Male Infant ^b^** | **49%** (21) | **51%** (22) | 0.83 |
| **Induction ^b^** | **7%** (3) | **35%** (15) | **0.0016** |
| **Mode of Delivery ^b^**  *NVD*  *ELCS*  *EMCS*  *INST.* | **65%** (28)  **16%** (7)  **7%** (3)  **12%** (5) | **40%** (17)  **21%** (9)  **23%** (10)  **21%** (9) | **0.020**  0.78  **0.041**  0.38 |
| **Pre-Term ^b^** | **0%** (0) | **26%** (11) | **0.00027** |
| **SGA ^b^**  **FGR ^b^** | **0%** (0)  **0%** (0) | **84%** (36)  **44%** (19) | **---** |
| **Stillborn ^b^** | **0%** (0) | **7%** (3) | --- |
| **Apgar <7 @ 5mins ^b^** | **0%** (0) | **9%** (4) | --- |
| **NICU ^b^** | **0%** (0) | **26%** (11) | ^---^ |

*NCC2 (AMA normal pregnancy outcome (NPO) vs adverse pregnancy outcome (APO)); n=43/group. Data are as mean (range) or percentage (number). IBC = individualised birthweight centile, NVD = normal vaginal delivery, ELCS = elective caesarean section, EMCS= emergency caesarean section, INST. = instrumental delivery. ^a^Mann-Whitney U Test or ^b^Fisher’s Exact test.* Significant differences are highlighted with **bold** p values.
